# Depression and stress are associated with latent profiles of problematic social media use among college students

**DOI:** 10.3389/fpsyt.2023.1306152

**Published:** 2023-11-30

**Authors:** Jingjing Cui, Yang Wang, Dongyu Liu, Haibo Yang

**Affiliations:** ^1^Faculty of Psychology, Tianjin Normal University, Tianjin, China; ^2^Faculty of Teacher Education, Zunyi Normal University, Zunyi, China; ^3^Key Research Base of Humanities and Social Sciences of the Ministry of Education, Academy of Psychology and Behavior, Tianjin Normal University, Tianjin, China; ^4^Tianjin Social Science Laboratory of Students’ Mental Development and Learning, Tianjin, China

**Keywords:** problematic social media use, college students, depression, anxiety, stress, latent profile analysis

## Abstract

**Background:**

The previous literature has demonstrated that depression, anxiety, and stress are significant predictors of problematic social media use. However, the manifestation of problematic social media use varies, and the predictive relationship between depression, anxiety, and stress with different subgroups of problematic social media use remains unclear. The aim of this research was to evaluate latent subgroups of problematic social media use among college students and to investigate the impact of depression, anxiety, and stress on these latent subgroups.

**Methods:**

A survey was carried out among college students in China using a cross-sectional approach. A total of 955 participants were included, with a mean age of 19.50 ± 1.22 years. Participants completed questionnaires containing the Bergen Social Media Addiction Scale (BSMAS) and the Depression, Anxiety, and Stress Scale-21 (DASS-21). The study employed latent profile analysis (LPA) to investigate latent subgroups of Chinese college students with problematic social media use, and a robust three-step approach was used to develop predictive regression mixed models of depression, anxiety, and stress on latent subgroups.

**Results:**

Problematic social media use of Chinese college students can be categorized into four latent subgroups, namely, the high-risk group, the moderate-risk with pleasure group, the moderate-risk with compulsion group, and the low-risk group. The regression model showed that there was a significant difference between the high-risk group and the low-risk group on the stress scale. There was a significant difference between the moderate-risk with pleasure group and the moderate-risk with compulsion group on the depression scale.

**Conclusion:**

Problematic social media use is heterogeneous, with depression and stress being potentially key factors influencing problematic social media use. Depression would make college students more likely to be moderate-risk with compulsion problematic social media users than moderate-risk with pleasure problematic social media users, and stress would make college students more likely to be high-risk problematic social media users than low-risk problematic social media users.

## 1 Introduction

### 1.1 Problematic social media use

The use of social media has become increasingly prevalent among college students, making it one of the most popular mobile applications today. It refers to a communication platform that enables users to engage with a broad or specific audience in real-time or delayed interactions, through which information can be created, commented and shared (1). QQ, WeChat, Weibo, and TikTok are the social media platforms that college students in China frequently utilize. College students can utilize social media anytime and anywhere without limitations due to the convenience and instant accessibility of smartphones (2, 3), which fulfills their spiritual desires and enhancing their overall life contentment and happiness (4–6). Nevertheless, problematic social media use can likewise result in various negative consequences (7–12). A previous study showed that problematic social media use was more prevalent among college students in mainland China (44.9%) compared to college students in other regions (the United States, Singapore, South Korea, and Japan) (13). Problematic social media use refers to an excessive preoccupation with social media, a strong drive to engage with it, and a significant expenditure of time and effort on social media at the expense of other social engagements, educational pursuits, professional commitments, interpersonal connections, as well as mental health and overall wellbeing (14, 15), and it belongs to a specific type of problematic Internet use (16). Although problematic social media use has numerous detrimental consequences like other addictive behaviors, the fifth edition of the *Diagnostic and Statistical Manual of Mental Disorders* (DSM-5) and the 11th revision of *International Classification of Diseases* (ICD-11) do not officially define problematic social media use as an addictive disorder. Presently, the prevailing consensus among scholars is that, although lacking formal recognition as an addictive disorder, the specific criteria pertaining to problematic social media use, namely clinical relevance, empirical evidence, and theoretical embedding, are congruent with the classification of “other specific addictive behavioral disorders” (code: 6C5Y) in the ICD-11 (17). Consequently, it is deemed appropriate to investigate this phenomenon within the framework of addiction (18, 19). The notion that problematic social media use may constitute a potential addictive disorder has been proposed by numerous scholars, and future versions of the DSM and ICD may be influenced by evidence-based discussions to potentially incorporate problematic social media use (20–22).

### 1.2 Classification of problematic social media use

The classification of problematic social media use has varied in previous studies. Some studies identified problematic and non-problematic users by setting clear boundaries (22). Some studies categorized problematic social media use into a non-addictive group, a problematic use group, and an addictive group by means (*M*) ± standard deviation (*SD*) (18). However, there is heterogeneity in social media use among college students, and previous variable-oriented methods have not taken into account a wide range of individual differences (21). Therefore, comprehending the simultaneous functioning of multidimensional personal characteristics within individuals and their role in creating diverse subgroups with different levels of problematic social media use is crucial (23). Person-oriented methods gather data at the personal level and have the ability to identify significant patterns of traits within subgroups (21). Latent profile analysis (LPA), as a person-oriented method, explains associations between exogenous variables in terms of the number of latent profiles. It can reveal latent subgroups with similar symptom profiles from the observed data, ensuring that between-group heterogeneity is maximized versus within-group heterogeneity is minimized, which will help researchers to understand the specific characteristics of each subgroup in a way that is unmatched by variable-oriented methods (24). The person-oriented method is especially suitable for categorizing diseases with diverse symptoms, as it can achieve a highly reliable classification by accurately discerning various characteristics. The application of latent profile analysis (LPA) has been utilized in studying problematic social media use and can provide an estimation of the percentage of individuals at varying degrees of vulnerability (24).

Many studies have found in latent profile analysis (LPA) that problematic social media use can be categorized into three latent subgroups, high-risk, moderate-risk, and low-risk, and what these studies have in common is that each of the latent subgroups scored roughly the same on each item (24–27). The aforementioned categorization results are similar to those of the variable-oriented method. However, it is flawed in its failure to highlight potential incongruous responses that certain groups may exhibit across different items. Some studies have found that some latent subgroups differ in their scores on each item. Luo et al. (28) distinguished between five latent subgroups, casual users and regular users who scored all six items on the Bergen Social Media Addiction Scale (BSMAS) as “very rarely” or “rarely,” and disordered users who scored all six items as “very often” or “often.” Although low-risk high-engagement users and at-risk high-engagement users scored similarly higher on the salience and tolerance criteria, low-risk high-engagement users scored much lower than at-risk high-engagement users on the mood modification, relapse, withdrawal, and conflict criteria. The results of this categorization reveal varying responses of certain group on different items. However, caution should be exercised due to the fact that one of the profiles represents less than 5% of the total population. The study also found that although the salience (the dominance of social media engagement in a person’s thoughts and everyday existence) and tolerance (the inclination to allocate increasing time on social media to attain the same level of enjoyment) criteria were highly endorsed, they were weak in distinguishing whether social media use was compulsive or not. Conflict (negative impact on work or school caused by inappropriate social media use), withdrawal (psychological distress when unable to access social media), and relapse (attempts to quit or control excessive social media use but failing to curb it) criteria provided considerable discriminatory power in identifying whether or not social media use was compulsive, and were strong independent predictors of problematic social media use (28, 29). From this, we hypothesized that some subgroups may have large differences in scores on conflict, withdrawal, and relapse indicators versus scores on salience and tolerance indicators. Therefore, an attempt can be made to classify problematic social media use into four latent subgroups. The first subgroup exhibited high scores on all items. The second subgroup displayed high scores on salience and tolerance items, with low scores on other items. The third subgroup demonstrated high scores on conflict, withdrawal, and relapse items, while scoring low on the remaining items. Lastly, the fourth subgroup obtained low scores across all items. This categorization avoids excessive classification while emphasizing the potential differential responses of these groups toward different aspects.

The Interaction of Person-Affect-Cognition-Execution (I-PACE) model proposed by Brand et al. provides a theoretical foundation for categorizing problematic social media use into four latent subgroups. It suggests that gratification is the primary, but not the only, driver of changes in affective and cognitive responses to Internet-related stimuli during the early stages of the process of specific problematic Internet use. Furthermore, it suggests that the degree of gratification diminishes as the addictive process advances, while the impact of compensation effects intensifies. Decreasing control over specific Internet applications can lead to an increase in negative experiences and emotions. The detrimental effects are worsened by the fact that the significance of gratification diminishes while the significance of compensation increases in the decision to repeatedly utilize Internet applications. The addictive process involves a transition from a voluntary and pleasurable to a habitual or compulsive pattern (16). Thus, there are two driving paths in the progression of problematic Internet use: “feels better” and “must do.” The path of “feels better” encompasses both positive (such as enjoyment and reward) and negative (such as decreased anxiety and unfavorable emotions) reinforcing experiences and related cravings and desires, which is the path linked to gratification. The path of “must do” might manifest at a later stage of the addictive process and encompasses habitual behaviors and even obsessive behaviors, which refer to maladaptive actions that persist despite being aware of their detrimental outcomes, and this path is linked to compensation (19). Therefore, the present study hypothesized that problematic social media users motivated by the “feels better” path are more likely to be “moderate-risk with pleasure” users with higher scores on salience and tolerance; that problematic social media users motivated by the “must do” path are more likely to be “moderate-risk with compulsion” users with higher scores on conflict, withdrawal, and relapse; and that high-risk problematic social media users with high scores on all six items are likely to be motivated by both paths. This leads us to propose the first hypothesis of the study, that as a specific type of problematic Internet use, problematic social media use may comprise four latent subgroups: high-risk, moderate-risk with pleasure, moderate-risk with compulsion, and low-risk.

### 1.3 Relationship between depression, anxiety, and stress and problematic social media use

Due to the negative impacts of problematic social media use, there is a great need to explore the risk factors of problematic social media use and their formation mechanisms. According to the I-PACE model, specific predisposing factors may be associated with different types of problematic Internet use (30). In the case of problematic social media use, mental distress (i.e., depression, anxiety and stress) emerges as a significant predisposing factor. Moreover, numerous studies have consistently reported a high prevalence of mental distress among college students (31). College students experiencing mental distress are more prone to developing problematic social media use. Mental distress has been indicated as a potential predictor of increased levels of problematic social media use (32–34). The reason why mental distress is related to problematic social media use may lie in the Internet’s compensatory function for the absence of reality, as suggested by Kardefelt-Winther’s compensation Internet use theory (CIUT) (35). According to this theory, people may turn to the Internet to alleviate their negative life situations and relieve their mental distress (36). Thus, mental distress can be viewed as an antecedent to Internet use, and excessive or heightened Internet use is perceived as a compensatory action to regulate mental distress, which, if uncontrolled, can lead to unregulated behaviors (37). Consequently, individuals experiencing depression, anxiety, or stress may allocate an imbalanced amount of time on social media as a means of managing their mental distress, despite the fact that this approach may not be beneficial for their wellbeing (38–40). Thus, mental distress may be a predictor of problematic social media use, and individuals experiencing depression, anxiety, and stress might favor virtual encounters over in-person interactions (34). However, previous research assessing the relationship between mental distress and problematic social media use has predominantly used total scores from measurement instruments (e.g., calculating total scores for the six addiction criteria and interpreting the severity of problematic social media use in terms of the total score) (41). As previously mentioned, problematic social media use among college students is heterogeneous, and there may be differences in the prediction of problematic social media use by depression, anxiety, or stress for different latent subgroups (42). Additionally, previous approaches might have concealed the potential diversity of these symptoms (41). Meanwhile, Billieux’s integrative pathway model (43) proposes that there are three main pathways that can lead to problematic smartphone use, a pathway of excessive reassurance, a pathway of impulsivity-antisocial, and a pathway of extraversion. Each of these pathways is linked to distinct risk factors. As a subtype of problematic smartphone use, exploring the distinct connections between the six addiction criteria of problematic social media use and the three forms of mental distress (depression, anxiety, and stress) can enhance comprehension of their correlation. The present study hypothesized that the pathways in which individuals experiencing depression, anxiety, or stress employ social media to manage mental distress, along with the resulting forms of problematic social media use, might vary.

Several research studies have indicated notable correlations between symptoms of depression and problematic social media use (26, 44). One study confirmed that depressive character can significantly and positively predict Facebook addiction (45). Another study using cluster analysis found that a cluster of adolescents with borderline personality adolescents disorder focused on depressive symptoms scored higher on problematic social media use than the normal group, and that depressive symptoms were found to be a valid predictive variable for problematic social media use (46). Depressed individuals often experience a diminished interest or enjoyment in nearly all activities (47). It has been suggested that these individuals typically engage in passive social media use, for example, scrolling through social media news, in an attempt to alleviate the depressive symptoms. However, they often express feelings of boredom and a lack of pleasure while using social media. This repetitive and joyless social media use can develop into a habitual or even compulsive behavior, where depressive symptoms automatically trigger passive social media use. Unfortunately, inhibiting this impulsive behavior becomes challenging (48). This is consistent with Brand’s description of the “must do” path (19), and thus, individuals with depression might have a higher tendency to develop “high compulsion” problematic social media use. Thus, we propose the second hypothesis of the study, that depression positively predicts moderate-risk with compulsion problematic social media use.

Research based on empirical studies has indicated that anxiety serves as a contributing factor to the development of problematic social media use (42, 49). Heightened levels of anxiety have been linked to excessive utilization of smartphones and a proclivity for sending an abundance of text messages (50). According to a study, individuals with anxiety may develop an overreliance on social media to promptly satisfy their psychological requirement for social solace. Their anxiety levels decrease when they utilize their smartphones to check notifications from family, friends, and loved ones (51). Experiencing prolonged anxiety generates a profound feeling of discomfort, prompting individuals to turn to social media as a means of compensating for their self-esteem, confronting their fears, and ensuring self-preservation. This strengthens the behavior of using social media, and ultimately, it leads to the development of problematic social media use (52). Some studies have confirmed that anxiety and fear of missing out are significantly correlated with problematic social media use, and that fear of missing out leads to the need to stay in frequent contact with social networks for comfort, therefore, the need for socialization is a key mechanism to explain problematic social media use and its association with anxiety (53). Anxious individuals attempt to relieve anxiety and gain pleasure through social media use, which is consistent with Brand’s description of the “feels better” path (19). Some researchers have also demonstrated that of the three subtypes of mental distress, only anxiety predicts low-risk high-engagement problematic social media use (as evidenced by high scores on salience and tolerance, and low scores on the other criteria) (41). Consequently, individuals experiencing anxiety might have a higher propensity to “high pleasure” problematic social media use. Therefore, we propose the third hypothesis of the study, that anxiety positively predicts moderate-risk with pleasure problematic social media use.

Numerous studies have indicated that stress is a positive predictor of problematic social media use (6, 54). According to Kardefelt-Winther’s findings, stress emerged as the sole influential factor for problematic Internet use when analyzing various psychological traits. The significance of loneliness and social anxiety in relation to excessive online gaming diminished when stress was taken into account, suggesting a greater stability of stress compared to other psychological traits (55). When college students perceive stress, they are inclined to seek out intense excitement and novel sensations and experiences, even if the situation contains danger and risk factors (56). It’s no wonder that individuals who are inclined toward excitement and enticed by incentives will frequently gravitate toward social media, considering its highly engaging nature and abundant options for amusement and enjoyment (23). Research consistently indicates that a propensity for sensation-seeking is linked to problematic social media use among late adolescents and adults (57). Additionally, a study proposed that social media attributes, such as socialization and entertainment services, enhance the perceived pleasure in a manner that reinforces uncontrolled social media use (58). In brief, it seems that the impact of stress on problematic social media use appear to be driven by both the “feels better” and “must do” paths. Some researchers have confirmed that stress only predicts high-risk problematic social media use (as evidenced by high scores on all six addiction criteria), and is not predictive of other subgroups of problematic social media use (41). Therefore, we propose the fourth hypothesis of the study, that stress positively predicts high-risk problematic social media use.

The study focused on college students and utilized the latent profile analysis technique to classify the problematic social media use among this group. It aimed to identify the various latent profiles of problematic social media use among college students and examine the connections between depression, anxiety, and stress with these different profiles.

## 2 Methods

### 2.1 Participants and procedure

A total of 1,188 college students were invited to participate in an online questionnaire at a university. However, 233 college students were excluded due to non-compliant responses on the polygraph questions, resulting in a receipt of 955 valid questionnaires. Of the 955 participants, 236 (24.70%) were male and 719 (75.30%) were female, aged 17–26 years old (*M* ± *SD* = 19.50 ± 1.22). Every participant willingly volunteered to take part in this research study and each participant duly signed a form indicating their informed consent. Participants were given the necessary emotional or psychological assistance when required. Ethical approval was granted by the Ethics Committee of Tianjin Normal University. This study adhered to the principles of the Declaration of Helsinki in its procedures.

### 2.2 Measures

#### 2.2.1 The Depression, Anxiety, and Stress Scale-21

The Depression, Anxiety, and Stress Scale-21 (DASS-21) is a revision by Antony et al. (59) of the Depression, Anxiety, and Stress Scale-42 (DASS-42) scale revised by Lovibond et al. (60). The measurement includes three subscales for measuring depression, anxiety, and stress, which eliminates the need for redundant assessments of these three conditions. Each subscale comprises 7 items, resulting in a total of 21 items for the entire measurement. Participants were asked to recall whether the corresponding symptoms had occurred within the previous week. They were then evaluated using a Likert scale with four points (0 representing non-compliance and 3 representing the highest level of compliance). Higher scores on the scale indicated a greater degree of mental distress. Multiple research studies have verified that the DASS-21 in its Chinese version exhibits a strong level of reliability and validity (31, 61–63).

#### 2.2.2 The Bergen Social Media Addiction Scale

In the field of addictive behaviors, while only gambling disorder and Internet gaming disorder have been officially recognized as addictive disorders so far, an increasing body of research also acknowledges problematic social media use as a potential addictive disorder. Therefore, it can be identified based on the six criteria of addiction (22). The Bergen Social Media Addiction Scale (BSMAS) (64) was derived from the Bergen Facebook Addiction Scale (BFAS) (22) by substituting the term “Facebook” with “Social Media.” The BSMAS is a commonly measurement for evaluating the symptoms and prevalence of problematic social media use in a population (64). The scale encompasses the six addiction criteria proposed by Griffiths, namely salience, tolerance, mood modification, withdrawal, conflict, and relapse, with each criterion being represented by one item, resulting in a six-item scale (29). Participants rated each item from 1 (rarely) to 5 (often) based on their personal encounters in the previous year. In the Chinese version of the BSMAS, social media is defined as “QQ, WeChat, Weibo, TikTok, etc.” (28), and the Chinese version of the BSMAS has been shown to have high reliability and validity (65).

### 2.3 Statistical analysis

Descriptive statistics were analyzed using SPSS 26.0 and common method bias was tested using Harman’s one-way test, if the maximum factor variance explained was <40%, it indicated the absence of serious common method bias (66).

A latent profile analysis (LPA) was conducted using M*plus* 8.3 (23), identifying subgroups of participants (latent profiles) who exhibited comparable responses on the Bergen Social Media Addiction Scale (BSMAS) items (i.e., similar levels of risk for developing problematic social media use). Fit indices of 1–6 profiles were extracted for model comparison using the robust maximum likelihood (RML) estimation.

To establish the number of latent profiles (ranging from 1 to 6), various criteria were employed, including information-theoretic methods, likelihood ratio statistical test methods, and the entropy-based criterion known as the Entropy index. The first method includes Akaike information criterion (AIC), Bayesian information criterion (BIC), and sample size-adjusted BIC (aBIC), where lower values indicate a leaner model and better model fit (67). The second method uses the Lo–Mendell–Rubin Likelihood Ratio Test (LMR-LRT) and the Bootstrap-based Likelihood Ratio Test (BLRT), and the *p* < 0.05 for both the LMR-LRT and BLRT indexes suggests that the model with k profiles fits better than the model with k-1 profiles (68). The third method comprises the last standard entropy value, ranging from 0 to 1. Higher values suggest improved distinction among profiles, and values above 0.8 indicate a classification accuracy exceeding 90%, with greater accuracy in classification (69). To avoid unrealistic solutions and overfitting, it is recommended that all latent profiles identified by the LPA consist of a minimum of 5% of the sample (25).

Regression mixed models were developed using a robust three-step approach based on M*plus*’ R3STEP program. Depression, anxiety, and stress were considered as independent variables, while the dependent variables were the latent profiles obtained in the initial stage. The aim was to account for the effects of these independent variables on the latent profiles and to validate the predictive role of depression, anxiety and stress on profile membership (70, 71).

## 3 Results

### 3.1 Common method bias test

In this study, the data was examined for common method bias using Harman’s one-way test. The unrotated exploratory factor analysis revealed five factors with a characteristic root greater than 1, and the maximum factor variance explained was 36.61% (<40%), so there was no serious common method bias in this study (66).

### 3.2 Latent profiles of problematic social media use

To explore latent patterns of social media use among college students, latent profiles were modeled based on participants’ means on each dimension of the Bergen Social Media Addiction Scale (BSMAS) (see Table 1).

**Table 1:** Summary of latent profile analysis.

No. of profiles	AIC	BIC	aBIC	Entropy	LMR-LRT	BLRT	Smallest profiles%
1	18246.16	18304.50	18266.39				
2	16938.98	17031.35	16971.00	0.82	<0.001	<0.001	49.6%
3	16703.23	16829.63	16747.06	0.77	0.017	<0.001	14.1%
4	16502.88	16663.32	16558.51	0.84	0.035	<0.001	18.1%
5	16347.85	16542.32	16415.28	0.85	<0.001	<0.001	5.2%
6	16223.21	16451.71	16302.44	0.84	<0.001	<0.001	7.0%

AIC, Akaike information criterion; BIC, Bayesian information criterion; aBIC, sample size-adjusted Bayesian information criterion; LMR-LRT, Lo–Mendell–Rubin Likelihood Ratio Test; BLRT, Bootstrap-based Likelihood Ratio Test.

The fitting indices AIC, BIC, and aBIC decelerated with increasing number of profiles. The deceleration of the fitting indices slowed down for the 3-profile model, but Entropy was equal to 0.77 (<0.80), with significant (*p* < 0.05) LMR-LRT and BLRT values; Entropy was equal to 0.84 (>0.80) for the 4-profile model, with significant (*p* < 0.05) LMR-LRT and BLRT values; Entropy was equal to 0.85 (>0.80) for the 5-profile model, with LMR-LRT and BLRT values were significant (*p* < 0.001), but one of the profiles accounted for 5.2% and the characteristics of the population in that profile were similar to the other profile. Considering the streamlining treatment of the model, this study proposed to categorize the participants into 4 profiles.

**Figure 1:**
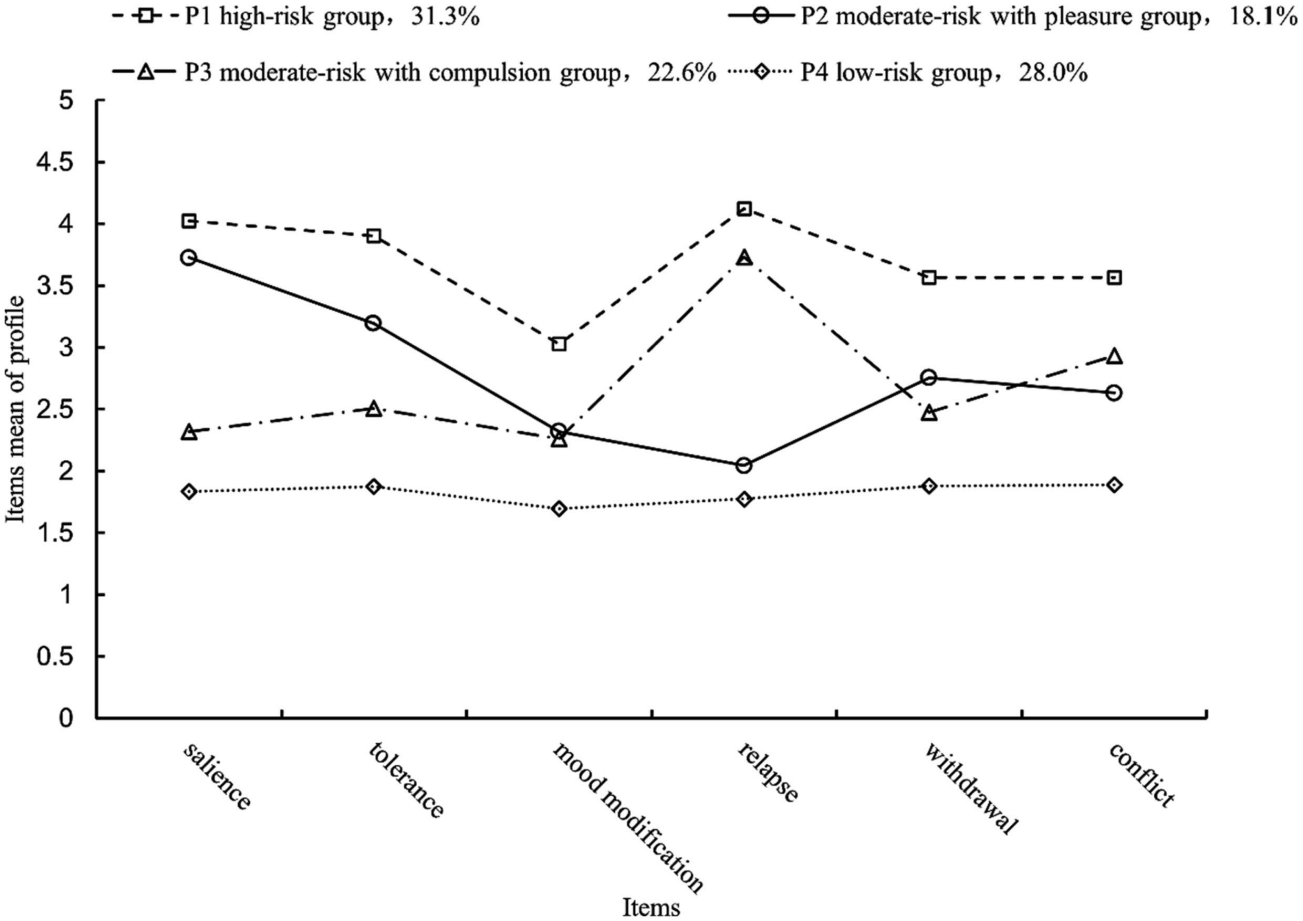
Mean values of latent profiles of problematic social media use.

The mean values of the 4-profile model on the six dimensions are shown in Figure 1. Based on Figure 1, it can be seen that profile 1 (31.3%) exhibited high mean values on the six dimensions of the Bergen Social Media Addiction Scale (BSMAS), all of which were greater than 3. This aligns with the criteria for addiction suggested by a previous study, which stated that at least four out of the six items should score 3 or higher (22). Therefore, profile 1 was named the “high-risk group” for problematic social media use. Profile 4 had a lower mean score of less than 2, so it was named the “low-risk group” for problematic social media use. Profile 2 had high means (>3) on the salience and tolerance dimensions and low means (<3) on the other dimensions. Salience and tolerance indicate the significant time and effort individuals dedicate to social media activities to derive pleasure, reflecting the important role of gratification in the early stages of addiction as described in the I-PACE model (16). Therefore, profile 2 was named as a “moderate-risk with pleasure group.” profile 3 had a high mean on the relapse dimension (>3) and a low mean on the other dimensions (<3). Relapse, in this context, refers to the unsuccessful attempt to quit or control the excessive use of social media, reflecting what is described in the I-PACE model, where compensation becomes increasingly important in the later stages of addiction and gradually changes from pleasure to compulsion to use. Profile 3 was therefore named the “moderate-risk with compulsion group.”

The scores on the Bergen Social Media Addiction Scale (BSMAS) showed significant differences among profile 1 (*M* = 22.28, *SD* = 3.00), profile 2 (*M* = 16.61, *SD* = 2.32), profile 3 (*M* = 16.27, *SD* = 2.30), and profile 4 (*M* = 10.85, *SD* = 2.70), *F*(3, 951) = 878.55, *p* < 0.001, $\eta_{p}^{2}=0.74$ . Multiple comparisons indicated significant differences between profiles (*p* < 0.001), except for profiles 2 and profiles 3 which were not statistically significant (*p* = 0.154). Although there was no statistically significant difference in the total score of the BSMAS between profiles 2 and profiles 3, profile 2 exhibited significantly higher scores than profile 3 on the salience and tolerance dimensions (*p* < 0.001), while significantly lower scores were observed for profile 2 compared to profile 3 on the relapse dimension (*p* < 0.001). The results suggest heterogeneity in problematic social media use.

### 3.3 Depression, anxiety, and stress in relation to problematic social media use

To examine the correlation between depression, anxiety, stress, and problematic social media use, we utilized latent profile analysis (LPA) results as the dependent variable. The independent variables consisted of the three subscales: depression, anxiety, and stress. By assessing the Odds Ratio (OR) values and their significance, we determined that the independent variables were predictive of the dependent variables when the OR values were significant. In this study, the low-risk group was used as the reference group, and there was a significant difference between the high-risk group and the low-risk group on the stress scale (*p* < 0.001). Furthermore, as participants’ stress scale scores increased, so did their likelihood of being part of the high-risk group (1.19 times). There were no significant differences between the moderate-risk with pleasure group and low-risk groups on any of the depression, anxiety, or stress scales, and there were no significant differences between the moderate-risk with compulsion group and low-risk groups on any of the depression, anxiety, or stress scales. Interestingly, there was a significant difference between the moderate-risk with pleasure group and the moderate-risk with compulsion group on the depression scale (*p* = 0.031), and the higher a participant’s score on depression, the lower the probability of belonging to the moderate-risk with pleasure group than to the moderate-risk with compulsion group (0.89 times lower). The results suggest that depression and stress can predict problematic social media use across different latent profiles (see Table 2).

**Table 2:** Odds ratio of problematic social media use among individuals with different levels of depression, anxiety, and stress.

Variables	P1 vs. P4		P2 vs. P4		P3 vs. P4		P2 vs. P3	
	OR	CI (95%)	OR	CI (95%)	OR	CI (95%)	OR	CI (95%)
Depression	1.03	0.95–1.12	0.98	0.88–1.08	1.09	1.00–1.20	0.89^*^	0.82–0.98
Anxiety	1.06	0.97–1.15	1.03	0.93–1.15	0.96	0.87–1.06	1.08	0.97–1.20
Stress	1.19^***^	1.10–1.30	1.10	1.00–1.21	1.09	0.99–1.19	1.01	0.92–1.10

P1, High-risk group; P2, Moderate-risk with pleasure group; P3, Moderate-risk with compulsion group; P4, Low-risk group; OR, odds ratio; CI, confidence interval. *p < 0.05, **p < 0.01, ***p < 0.001.

## 4 Discussion

### 4.1 Latent profiles of problematic social media use

By employing latent profile analysis, the research examined four subgroups of college students’ problematic social media use: high-risk group (31.3%), moderate-risk with pleasure group (18.1%), moderate-risk with compulsion group (22.6%), and low-risk group (28.0%). Hypothesis 1 was supported. The study confirms previous research that college students are heterogeneous in their problematic social media use (24, 26). Previous research has classified individuals into three subgroups (high-risk, moderate-risk, and low-risk) (25, 27). The present study found that different subgroups could be separated from the moderate-risk group, i.e., the moderate-risk with pleasure group and the moderate-risk with compulsion group. It was also observed that the moderate-risk with pleasure group may be driven more by the “feels better” path and the moderate-risk with compulsion group may be driven more by the “must do” path, while the high-risk group is equally driven by both paths (19). The findings offer empirical evidence supporting the I-PACE model, and the study confirms that problematic social media use is influenced by two factors: gratification and compensation. Gratification corresponds to the “feels better” path, characterized by a voluntary and pleasurable process. On the other hand, compensation corresponds to the “must do” path, which denotes a habitual and compulsive process (16). Many addictive disorders involve a transition from pleasure to compulsion, and this same transition could potentially occur in the case of problematic social media use. However, it is important to note that high-risk problematic social media users may encounter heightened levels of both pleasure and compulsion (72). This research partially addresses the issue of insufficient specificity in classifying problematic social media use in prior studies.

### 4.2 Depression, anxiety, and stress predict latent profiles of problematic social media use

According to the current research, college students who experience depression were more inclined to be moderate-risk with compulsion problematic social media users than moderate-risk with pleasure problematic social media users. Hypothesis 2 was supported. The moderate-risk with compulsion group obtained higher scores on the relapse criterion, and the criteria of conflict, withdrawal, and relapse have been widely acknowledged as fundamental elements of addiction (73). Some studies have shown that these criteria have strong discriminatory power in identifying whether or not one is a compulsive problematic social media user (28). This is consistent with previous findings, where it was found that depression levels significantly predicted total Internet Addiction Test (IAT) scores among college students (74), and that depression significantly and positively predicted problematic social media use (45). Since the core symptom of moderate-risk with compulsion problematic social media use manifests as relapse, i.e., diminished control over use. According to Billieux’s integrative pathway model (43), depression’s prediction of moderate-risk with compulsion problematic social media use may be realized through the impulsive-antisocial pathway. This pathway suggests that addictive usage patterns are linked to particular impulsive characteristics. Numerous studies have consistently shown a strong link between depression and impulsive characteristics (75, 76), and that impulsiveness positively predicts problematic social media use (77, 78). Several research studies have verified that there is a connection between depression and impulsive compulsive behaviors like addictive disorders, where impulsiveness acts as a mediator (79, 80). This evidence may explain why depression in college students leads to impaired impulse control and ultimately produces moderate-risk with compulsion problematic social media use.

As with previous findings (39, 41), this study discovered that stress increases the likelihood of college students becoming high-risk problematic social media users. Hypothesis 4 was supported. Previous studies have demonstrated a positive correlation between stress levels and the development of problematic social media (54). Young et al. (81) argued that in Chinese culture, students are under great academic pressure in secondary school, and when they enter university, they will seek happiness through social media, which will lead to addiction in the long run. High-risk problematic social media users who score greater than 3 on all six criteria of addiction not only experience fun and excitement in social media, but also have difficulty controlling their use. According to Billieux et al. (43) integrative pathway model, the prediction of stress on high-risk problematic social media use may be realized through the extroversion pathway, which hypothesizes that highly extroverted individuals who seek sensations and rewards are prone to developing addictive patterns of use. The stress-buffering hypothesis posits that individuals with elevated levels of stress are more prone to exhibit heightened levels of sensation-seeking (82). One study found that people who scored high on aspects of the Behavioral Activation System (i.e., drive, reward responsiveness, fun seeking) were prone to smartphone addiction (23). Research on factors that contribute to addiction vulnerability indicates that sensation-seeking is a significant predictor of addictive behavior (56). The provided evidence suggests that college students who experience stress are more inclined to seek pleasure, which subsequently leads to the development of compulsive usage patterns. Consequently, this engenders high-risk problematic social media use.

Contrary to prior studies, the current investigation failed to uncover any indication of anxiety predicting problematic social media use (49, 51). Nonetheless, there have been limited investigations indicating comparable outcomes, and one particular study revealed that individuals with elevated scores on the depression and stress scales also exhibited higher scores on problematic Internet use. Notably, no significant association was observed between anxiety and problematic Internet use (83, 84). Furthermore, a 3-year longitudinal study also showed that the development of future problematic smartphone use was not foretold by anxiety (85). Anxious individuals are reluctant to participate in social activities, show lower social interaction expectations, and even social withdrawal. They may resort to avoiding social media use to alleviate distress, e.g., paying less attention to the updated status of others’ social media, checking notifications of new information less often, or using other non-social features. Thus, people with high anxiety do not rely on smartphones perhaps due to a lack of social motivation (86). van Deursen et al. (87) found that anxiety was more closely related to process-oriented smartphone use than to social-oriented use. This finding supports social avoidance theory (88) and safety behavior theory (89), suggesting that anxiety can result in avoiding social-oriented smartphone use and instead opting for more process-oriented smartphone use, due to avoidance of social interactions and maintenance of safety behaviors. In the context of compensation Internet use theory (CIUT), anxiety should specifically drive the choice of process-oriented smartphone use as an alternative to choosing social media (90). People experiencing social anxiety desire social connections but are afraid of engaging in social activities, leading them to frequently avoid or reduce interactions due to perceiving them as challenging and uncomfortable (91). The act of avoiding social interactions can be demonstrated by avoiding in-person interactions or by using non-social functions on smartphones instead of engaging in online social media activities (92, 93).

The findings highlight how different latent profiles of problematic social media use are driven by different pathways supported by different psychological processes, as well as emphasizing how depression and stress lead to various latent profiles of problematic social media use. These findings are consistent with the predictions of compensation Internet use theory (CIUT). According to this theory, individuals with depression and stress are more prone to developing problematic social media use. This is because depression and stress compel individuals to resort to social media as a coping mechanism, which in turn leads to unhealthy patterns of social media use (35). Additionally, our findings offer partial validation of the integrative pathway model of problematic smartphone use at the compensatory pathways level. This model suggests that individuals have varying tendencies toward problematic smartphone use (43). According to this model, there are three primary pathways leading to problematic smartphone use, an excessive reassurance pathway, an impulsive-antisocial pathway, and an extraversion pathway. Each pathway is linked to distinct risk factors. Specifically, the present study confirmed the model in problematic social media use. First, according to the extraversion pathway, individuals with a strong inclination toward extraversion and a desire for sensation and reward are prone to problematic social media use. The extraversion pathway applies to individuals whose problematic social media use is characterized by obsessive-compulsive symptoms, attraction to exciting stimuli, or a desire to form new relationships with others. This aligns with the high-risk problematic social media use observed in this study. Second, according to the impulsive-antisocial pathway, problematic social media use is linked to distinct impulsive characteristics, such as a sense of urgency (a proclivity to hastily respond in emotional circumstances), a lack of planning (a proclivity to ignore consequences), or diminished self-control (a propensity to behave automatically rather than with restraint). This pathway is for individuals who are motivated by a lack of self-control, leading to uncontrollable impulsive actions and excessive use of social media. This aligns with the moderate-risk with compulsion problematic social media use observed in this study. Finally, a possible reason why our study did not validate the hypothesis of the excessive reassurance pathway is that the model was originally designed for smartphones, which have other non-social features in addition to social features. It is plausible that individuals with anxiety tendencies are more prone to utilizing the non-social aspects of their smartphones, making the pathway more relevant to other smartphone applications rather than social media.

### 4.3 Research contribution

First, the current investigation expands prior studies on problematic social media use by utilizing mixed modeling or person-oriented method, allowing for the identification of different latent subgroups and significant distinctions among them. Furthermore, these latent profiles are supported by the I-PACE model (19, 27). Second, our results align with the integrative pathway model of problematic smartphone use, positing that people have varying inclinations toward problematic smartphone use (43). The current research validates the existence of two primary pathways that contribute to problematic social media use, namely the impulsive-antisocial pathway and the extraversion pathway, both of which are linked to distinct risk factors.

The results of this research also carry significance for application, as effective measures to prevent or address problematic social media use should focus on the particular psychological aspects that motivate such behavior. First, stress may result in high-risk problematic social media use among college students. Hence, it is imperative for universities to strengthen students’ capacity to deal with pressure, encompassing instruction in emotional intelligence and stress management, alongside tailored coursework to enhance cognitive capabilities. This approach aims to deter students from resorting to excessive social media use as a means of evading stress (94). Second, depression can lead to moderate-risk with compulsion problematic social media use among college students. Hence, it is imperative for universities to prioritize the issue of depression among college students and offer consistent counseling services to individuals experiencing elevated levels of depression, with the aim of mitigating their depressive symptoms. In order to decrease emotional insecurity and reliance on problematic social media use, individuals experiencing severe depression may require increased social support to enhance their mood (23).

## 5 Limitations

Using the latent profile analysis (LPA) method, this research examined the diversity within college students’ problematic social media use. The research classified problematic social media use into four different latent subgroups and investigated the factors that make each subgroup more prone to such behavior. The study addressed certain limitations of the variable-oriented methods to a certain extent, but several shortcomings remain: Firstly, data collection in this study solely relied on the self-assessment questionnaire method, which inevitably introduced the social praise bias. In future research, methods such as the other-assessment method or the experimental method can be used in a comprehensive manner in order to obtain more objective results. Secondly, this study solely relied on a cross-sectional design, making it impossible to establish a causal relationship between the variables. To further substantiate the findings of this research, future studies should consider employing longitudinal or experimental approaches. Furthermore, it is important to highlight that latent profile analysis (LPA) is primarily an exploratory technique. The determination of the number of latent profiles relies on the assessment of fit indices, interpretability, and utility, which can potentially lead to misclassification. It should be validated in future studies in conjunction with clinical diagnosis.

## 6 Conclusion

Problematic social media use is heterogeneous, problematic social media use among Chinese college students can be categorized into four latent subgroups, namely, the high-risk group, the moderate-risk with pleasure group, the moderate-risk with compulsion group, and the low-risk group. Problematic social media use may be influenced by depression and stress, which are considered as significant factors. College students experiencing depression are more prone to being moderate-risk with compulsion problematic social media users rather than moderate-risk with pleasure problematic social media users, while stress increases the likelihood of college students being high-risk problematic social media users instead of low-risk problematic social media users. In conclusion, distinct forms of social media use might be linked to particular forms of distress.

## Data availability statement

The original contributions presented in the study are included in the article/supplementary material, further inquiries can be directed to the corresponding author.

## Ethics statement

The studies involving humans were approved by the Ethics Committee of Tianjin Normal University. The studies were conducted in accordance with the local legislation and institutional requirements. The participants provided their written informed consent to participate in this study.

## Author contributions

JC: Conceptualization, Data curation, Formal analysis, Methodology, Writing – original draft, Writing – review & editing. YW: Data curation, Formal analysis, Investigation, Writing – review & editing. DL: Data curation, Writing – review & editing, Investigation. HY: Conceptualization, Funding acquisition, Validation, Writing – review & editing.
